# Ultrasensitive and Simple Dopamine Electrochemical Sensor Based on the Synergistic Effect of Cu-TCPP Frameworks and Graphene Nanosheets

**DOI:** 10.3390/molecules28062687

**Published:** 2023-03-16

**Authors:** Liudi Ji, Qi Wang, Xiaqing Gong, Jiamin Chen, Xiaoming Zhu, Zeyu Li, Peng Hu

**Affiliations:** 1 Hubei Key Laboratory of Radiation Chemistry and Functional Materials, Non-Power Nuclear Technology Collaborative Innovation Center, Hubei University of Science and Technology, Xianning 437100, Chinazhuxiaoming@hbust.edu.cn (X.Z.); 2 School of Pharmacy, Hubei University of Science and Technology, Xianning 437100, China

**Keywords:** 2D MOFs, graphene, solvent exfoliation, electrochemical determination, enhancement mechanism

## Abstract

Dopamine (DA) is an important neurotransmitter. Abnormal concentration of DA can result in many neurological diseases. Developing reliable determination methods for DA is of great significance for the diagnosis and monitoring of neurological diseases. Here, a novel and simple electrochemical sensing platform for quantitative analysis of DA was constructed based on the Cu-TCPP/graphene composite (TCPP: Tetrakis(4-carboxyphenyl)porphyrin). Cu-TCPP frameworks were selected in consideration of their good electrochemical sensing potential. The graphene nanosheets with excellent conductivity were then added to further improve the sensing efficiency and stability of Cu-TCPP frameworks. The electrochemical properties of the Cu-TCPP/graphene composite were characterized, showing its large electrode active area, fast electron transfer, and good sensing performance toward DA. The signal enhancement mechanism of DA was explored. Strong accumulation ability and high electrocatalytic rate were observed on the surface of Cu-TCPP/graphene-modified glassy carbon electrode (Cu-TCPP/graphene/GCE). Based on the synergistic sensitization effect, an ultrasensitive and simple DA electrochemical sensor was developed. The linear range is 0.02–100 and 100–1000 µM, and the detection limit is 3.6 nM for the first linear range. It was also successfully used in detecting DA in serum samples, and a satisfactory recovery was obtained.

## 1. Introduction

Dopamine (DA) is a vital neurotransmitter. It acts a crucial role in the regulation of the central nervous system and participates in almost all physiological functions [1]. Abnormal concentration of DA can result in many neurological diseases, including Parkinson’s syndrome, Alzheimer’s diseases, and schizophrenia [2,3]. Therefore, the simple and convenient determination of DA means a lot for the diagnosis and monitoring of neurological diseases. In recent years, researchers have made a great deal of effort around the development of high sensitivity and selectivity determination techniques of DA. Spectrometric techniques [4], high-performance liquid chromatography–diode array (HPLC-DA) [5], fluorometry [6], and chemiluminescence method [7] have all been reported for the detection of DA. However, most of these methods are complicated to operate, high cost, and time consuming. Beyond that, electrochemical methods are attractive in the determination of DA because of their low cost, user-friendly control, fast determination, high sensitivity, and ability to enable miniaturization [8,9]. Many nanomaterials with different functions have been used to construct the modified electrode, such as g-C_3_N_4_/MWNTs/GO [10], Cu_x_O-ZnO/PPy/RGO [11], Fe_2_O_3_-NiO@GO [12], and f-MWCNTs/GO/AuNPs [13]. However, there is still much room for improvement in the electrode modification materials, for example, the simplicity, sensitivity, and selectivity. The development of enzyme-based biosensors has provided an important helping hand to improve these problems. For example, laccase, a kind of multicopper oxidase enzyme, has been successfully used to detect DA [14,15,16]. However, the high cost, poor stability, and low electron transport efficiency limit its practical application. Therefore, novel and high-performance electrode sensitive materials must be designed and synthesized.

Metal–organic frameworks (MOFs) are novel multifunctional porous materials and formed through coordination bonds by the self-assembly of organic ligands and metal ions or clusters. They have many excellent structural characteristics, for instance, abundant pore structure, large surface area, and unsaturated metal sites, which make MOFs have good potential for electrochemical sensing applications [17,18]. However, the MOFs used in electrochemical sensors are mainly 3D bulk crystals. Their big sizes or thicknesses inhibited the performance to some extent. Compared with 3D bulk MOFs, 2D MOF nanosheets exhibit ultrathin thickness and more accessible active sites, resulting in large surface area, fast electron transfer, easy mass transport, and high electrocatalytic ability [19,20,21]. Moreover, the poor conductivity of MOFs also reduces their property. The recombination with suitable conductive additives is needed to realize their full potential synergistically [22,23]. 

**Scheme 1:**
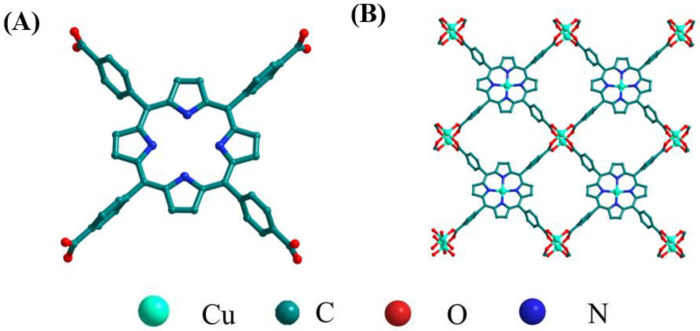
Structures of H_2_TCPP (**A**) and Cu-TCPP frameworks (**B**).

In this work, Cu-TCPP frameworks and graphene nanosheets were synthesized through solvent exfoliation in N,N-dimethylformamide (DMF) and N-methyl-2-pyrrolidone (NMP), respectively. Further, Cu-TCPP frameworks were composited with graphene nanosheets using a simple mixing technique. Here, Cu-TCPP is a typical and more studied 2D MOF. As displayed in Scheme 1, TCPP, as a large ring molecule with symmetric plane (A), can build a paddlewheel secondary building unit (SBU) with two copper metal cations (B). The paddlewheel SBU has a planar quadrilateral configuration and is an ideal SBU for creating 2D MOFs [24]. The selection of graphene nanosheets is because of their excellent conductivity and high stability. Moreover, the introduction of graphene nanosheets between 2D Cu-TCPP nanosheets can reduce the self-restacking of 2D MOFs by forming a structure with alternating MOF/graphene layers. Such a Cu-TCPP/graphene composite is thus expected to show superior sensing properties. Various techniques were used for characterizations, and then its sensing performance toward voltametric monitoring of DA was performed using differential pulse voltammetry (DPV). This method realized the simple and fast determination of DA with a wide linear range and low detection limit. Finally, this sensor was successfully used in serum samples with satisfactory recovery. 

## 2. Results and Discussion

### 2.1. Characterization of Cu-TCPP/Graphene Composite

**Figure 1:**
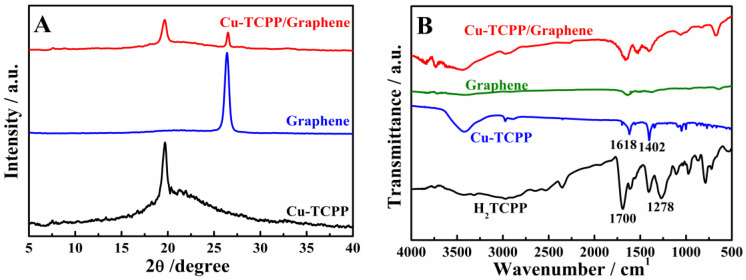
XRD pattern (**A**) and FT-IR spectra (**B**) of the prepared materials.

XRD was applied to investigate the composition and crystal structures of the obtained materials (Figure 1A). The specific diffraction peak at 7.5° and 19.6° could correspond to the (110) and (002) crystal plane of Cu-TCPP nanosheets [25]. For the graphene nanosheets, a strong peak at approximately 26.58° was assigned to the (002) plane of graphite [23]. The XRD pattern of Cu-TCPP/graphene exhibits all the three typical peaks, indicating that the obtained composite consisted of the Cu-TCPP framework and graphene. 

To further understand the bonding properties of the as-prepared materials, their FT-IR spectroscopy (Figure 1B) was performed. The FT-IR spectrum of H_2_TCPP (curve a) has two typical absorption bands at 1700 cm^−1^ and 1278 cm^−1^, which are attributed to the C=O stretching band. For Cu-TCPP nanosheets, two new bands at 1618 cm^−1^ and 1402 cm^−1^ representing the OC-O-Cu bond are detected [25,26,27], indicating the coordination of copper centers with organic ligands and the formation of Cu-TCPP. The spectrum of graphene confirms the existence of C=C stretching at 1620 cm^−1^ [28]. Furthermore, for the Cu-TCPP/graphene composite, the characteristic absorption peaks of Cu-TCPP and graphene all appear, confirming the successful combination of Cu-TCPP nanosheets and graphene.

**Figure 2:**
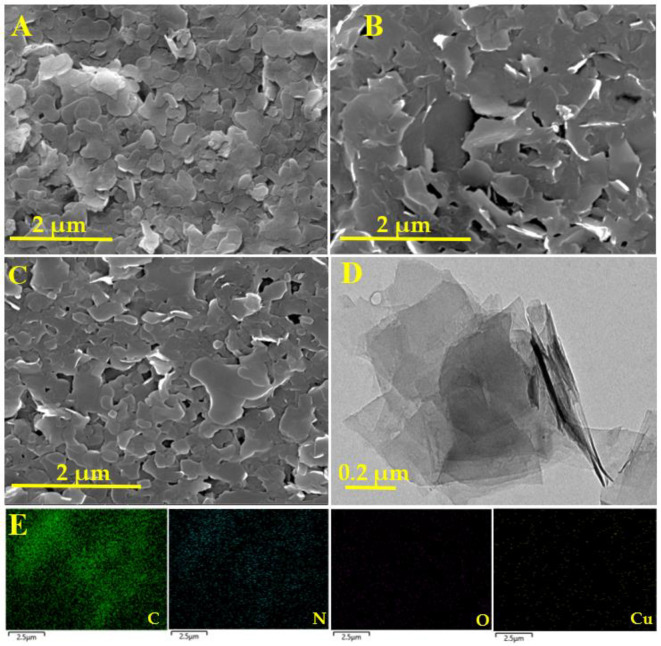
SEM images of Cu-TCPP (**A**), graphene (**B**), and Cu-TCPP/graphene composite (**C**); (**D**) TEM image of Cu-TCPP/graphene hybrid; (**E**) elements mapping images of Cu-TCPP/graphene hybrid.

Then, SEM was selected to check the morphological features of the prepared Cu-TCPP, graphene, and the Cu-TCPP/graphene composite. The Cu-TCPP shows a sheet-like morphology (Figure 2A). Its average diameter is approximately 500 nm. From Figure 2B, large graphene flakes are observed. After mixing, it is noticed that the Cu-TCPP nanosheets with smaller size spread among the graphene flakes (Figure 2C), suggesting the successful composite of Cu-TCPP and graphene nanosheets. Additionally, a layer structure is observed in the TEM image of the Cu-TCPP/graphene composite (Figure 2D). Some graphene nanosheets are stacked together and folded up, and quasi-circular Cu-TCPP nanosheets with smaller size are intercalated, further indicating the formation of the unique hierarchical hybrids. The stable intercalated structure maybe mainly caused by π-π stacking. Furthermore, the elemental mapping analysis reveals the homogeneous distribution of C, N, O, and Cu elements (Figure 2E), reconfirming the successful synthesis of the Cu-TCPP/graphene hybrid.

### 2.2. Electrochemical Properties of Cu-TCPP/Graphene Composite

**Figure 3:**
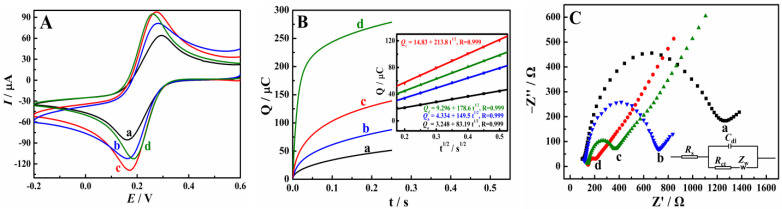
CV behaviors (**A**) and *Q*-t curves (**B**) of different GCEs in 5.0 mM K_3_[Fe(CN)_6_] (1.0 M KCl), inset: *Q*-t^1/2^ plots; (**C**) Nyquist impedance plots of different GCEs in 5 mM K_3_/K_4_Fe(CN)_6_ (0.1 M KCl). The measurements were carried out at the open circuit potentials. *R*_s_, *R*_ct_, *C*_dl_, and *Z*_w_ represent the solution resistance, the electron transfer resistance, the double-layer capacitance, and Warburg impedance, respectively. The used electrodes are GCE (a), Cu-TCPP/GCE (b), Cu-TCPP/graphene/GCE (c), and graphene/GCE (d).

Using potassium ferricyanide (K_3_[Fe(CN)_6_]) as the probe, its CV behavior was studied to explore the electrochemical properties of different GCEs (Figure 3A). A pair of redox peaks were observed on both GCEs. However, the peak currents and potentials are varied. For the Cu-TCPP/GCE (curve b), graphene/GCE (curve d), and Cu-TCPP/graphene/GCE (curve c), the current signals increase in sequence, indicating an increased active area. Furthermore, it is worth noting that the peak potential separations decrease gradually on Cu-TCPP/GCE, Cu-TCPP/graphene/GCE, and graphene/GCE, suggesting the enhanced electron transfer ability. 

Then, the electrochemical active area and electron transfer ability of different GCEs were further compared. Firstly, the chronocoulometry technique was applied to calculate the effective electrochemical area of the bare GCE, Cu-TCPP/GCE, graphene/GCE, and Cu-TCPP/graphene/GCE. The curves of charge (*Q*)-time (*t*) of different GCEs in 5.0 mM K_3_[Fe(CN)_6_] (1.0 M KCl) are provided in Figure 3B and converted to *Q*-*t*^1/2^ straight lines (the inset of Figure 3B). According to the Cottrelle equation [29]:

*Q* = 2nFAC*D^1/2^ π^−1/2^
*t*^1/2^ + *Q*_dl_ + *Q*_ads_ (1)

where *n* is the electron transfer number (*n* = 1), *F* is the faraday constant (96,485 C mol^−1^), C* and *D* are the initial concentration (5 mM) and diffusion coefficient (7.6 × 10^−6^ cm^2^ s^−1^) of K_3_[Fe(CN)_6_], and the active area (*A*) is directly obtained to be 0.055, 0.100, 0.112, and 0.143 cm^2^ for GCE, Cu-TCPP/GCE, graphene/GCE, and Cu-TCPP/graphene/GCE based on the slope of the linear equations in the inset of Figure 3A.

Furthermore, the electron transfer abilities of different GCEs were examined by electrochemical impedance spectroscopy (EIS) using 5 mM [Fe(CN)_6_]^3−/4^ as probe molecules, and the Nyquist plots are shown in Figure 3C. Based on the Randles equivalent circuit (the inset in Figure 3C), the charge-transfer resistance (*R*_ct_) on GCE (curve a), Cu-TCPP/GCE (curve b), graphene/GCE (curve d), and Cu-TCPP/graphene/GCE (curve c) are fitted to 1143, 604.2, 90.84, and 258.6 Ω from the diameter of semicircles. The smaller *R*_ct_ represents its better electron transfer ability. The results manifest that the combination of graphene effectively facilitates the electron transfer of Cu-TCPP.

**Figure 4:**
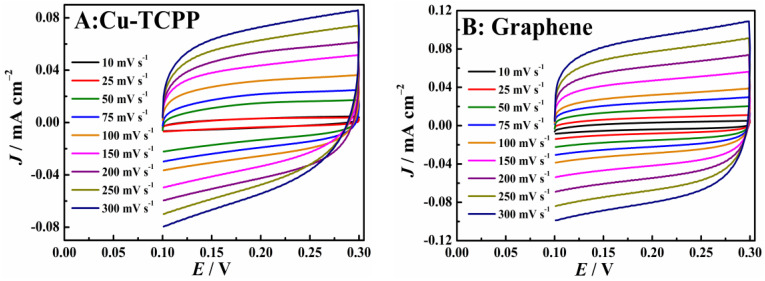

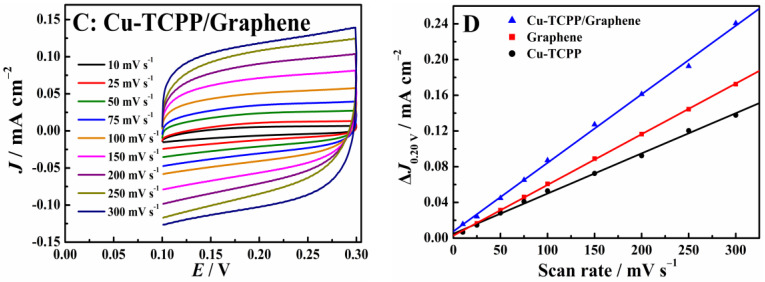
(**A**–**C**) CV curves of different materials with varied rates in 1 M KOH; (**D**) capacitive current at 0.20 V vs. scan rates.

Then, the electric double-layer capacitance (*C*_dl_) of the obtained materials was calculated to further compare the electrochemical active area. According to the reported experimental results [30], the exposed copper active sites in Cu-TCPP exit some redox waves in the region of 0.1 to −0.6 V. Therefore, the CV curves in a non-Faradaic region (e.g., from 0.10 to 0.30 V) were recorded under different scan rates (Figure 4A–C). The *C*_dl_ of Cu-TCPP, graphene, and Cu-TCPP/graphene was obtained to be 0.450 mF cm^−2^, 0.568 mF cm^−2^, and 0.768 mF cm^−2^ based on the slope of the linear equations between the scan rates and the capacitive currents (∆*J*) at 0.20 V (Figure 4D) [31,32,33]. The large *C*_dl_ also represents a large electrochemical active area. Clearly, the Cu-TCPP/graphene composite owns the largest active area. The results are consistent with the above results. In general, the Cu-TCPP/graphene composite owns excellent electrochemical properties and is suitable for the construction of the electrochemical sensing platform. 

### 2.3. Signal Enhancement Mechanism for DA on Cu-TCPP/Graphene/GCE

**Figure 5:**
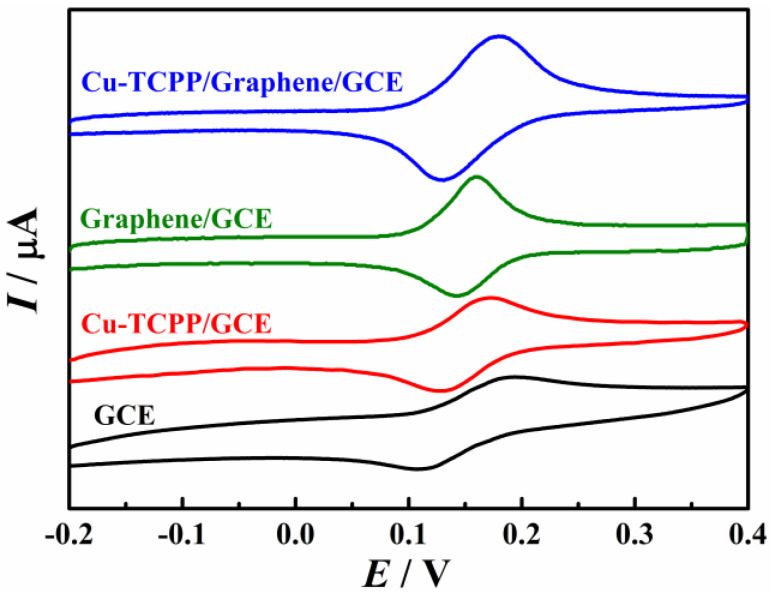
CV curves of different GCEs in 100 µM DA in pH 7.0 PBS.

The electrochemical behaviors of DA on different GCEs were first examined using CV. As shown in Figure 5, a pair of redox peaks are all observed on both four GCEs. However, the peak currents and peak potentials are different. On the surface of bare GCE, the redox peak current is small, and the peak potentials of oxidation and reduction are 0.189 and 0.115 V, respectively. Upon modifying Cu-TCPP frameworks on the surface of GCE, the currents start to increase. Meanwhile, the oxidation peak shifts negatively to 0.179 V, and the reduction peak shifts positively to 0.129 V, showing a high catalytic ability toward the oxidation of DA. For graphene/GCE, the above phenomenon is more obvious, which may have benefited from the electrochemical reactivity and excellent conductivity of graphene. Interestingly, the electrochemical responses of DA were further enhanced on Cu-TCPP/graphene/GCE, indicating typical synergistic effects. 

**Figure 6:**
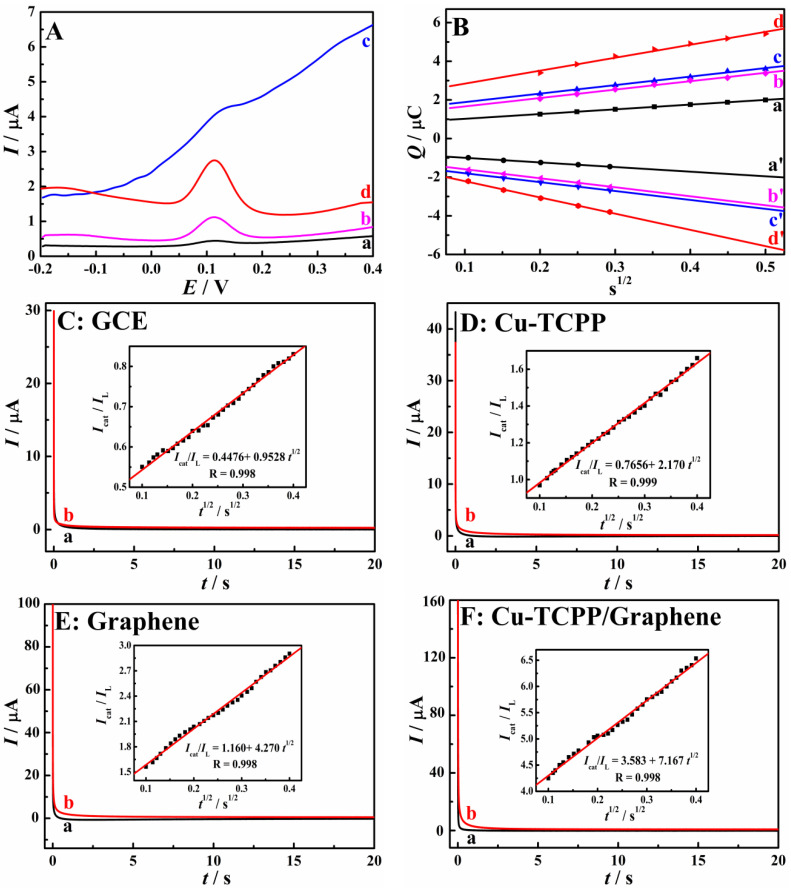
(**A**) DPV curves of 1 µM DA in pH 7.0 PBS on different GCEs; (**B**) plots of *Q*_f_ vs. *t*^1/2^ during the forward step (a–d) and *Q*_r_ vs. *f*(*t*) during the reverse step (a′–d′) on GCE (a and a′), Cu-TCPP/GCE (b and b′), graphene (c and c′), and Cu-TCPP/graphene/GCE (d and d′); (**C**–**F**) chronoamperometry behaviors on different GCEs in the absence (a) or presence of 1 mM DA in pH 7.0 PBS, inset: plots of *I*_cat_/*I*_L_-*t*^1/2^.

Subsequently, differential pulse voltammetry (DPV) was further used to confirm the different electrochemical activity of DA with relatively low concentration (1 µM) on the surface of different materials. As shown in Figure 6A, a small oxidation peak at 0.112 V is found on the surface of GCE (curve a), indicating the poor oxidation activity of DA. The oxidation signal on the Cu-TCPP/GCE is increased to some extent (curve b), which may result from the abundant active sites and large surface area of Cu-TCPP. When graphene nanosheets are modified on the surface of GCE (curve c), a higher background current is observed, which may be due to its excellent conductivity. The largest oxidation signal is obtained on the Cu-TCPP/graphene/GCE (curve d), suggesting the best properties of the Cu-TCPP/graphene composite toward electrochemical sensing of DA. The results further confirm the synergistic effect between Cu-TCPP and graphene nanosheets.

In order to explore the reason for the signal enhancement effects for the oxidation of DA, the double potential step chronocoulometry was applied to study its adsorption behaviors on different GCEs. First, the charge (*Q*)-time (*t*) curves in 7.0 PBS containing 1 µM DA were individually recorded. In the forward (positive) step (−0.2 V to 0.4 V), the charge (*Q*_f_) is indicated as [29]:

(2) $$Q_{f}=2nFAC^{*}D^{1/2}\pi^{-1/2}t^{1/2}+Q_{\mathrm{dl}}+Q_{\mathrm{ads}}$$

For the reverse step (0.4 V to −0.2 V), the charge (*Q*_r_) is defined by [29]:

(3) $$Q_{r}=2nFAC^{*}D^{1/2}\pi^{-1/2}f(t)+Q_{\mathrm{dl}}$$

(4) $$f(t)=\tau^{1/2}+{\left(t-\tau \right)}^{1/2}-t^{1/2}$$

where *Q*_dl_ and *Q*_ads_ represent the double-layer charge and the Faradaic charge of the oxidation of the adsorbed species, respectively. Therefore, the intercept of the plot of *Q*_f_-*t*^1/2^ (a–d) is the summation of *Q*_dl_ and *Q*_ads_. For the reverse step, the intercept of the *Q*_r_-*f*(*t*) plot (a′–d′) is *Q*_dl_. From Figure 6B and Table 1, the *Q*_ads_ of DA on different GCEs are easily obtained. Here, the values of *Q*_ads_ for DA on the surface of GCE, Cu-TCPP/GCE, graphene/GCE, and Cu-TCPP/graphene/GCE are calculated to be 0.0246, 0.106, 0.115, and 0.408 μC. The significantly increased *Q*_ads_ value indicates a higher accumulation efficiency of DA on the surface of Cu-TCPP/graphene composite, which may be attributed to the abundant adsorption sites and high conductivity.

Then, the electrochemical kinetics experiment was performed using the chronoamperometry technique to explore the obviously increased oxidation signals further. The *I*-*t* plot of different GCEs in 7.0 PBS (a) and in the presence of 1 mM DA (b) are recorded in Figure 6C–F. Then, the apparent catalytic rate constant (*k*_cat_) can be obtained according to the bottom equation [29]:

*I*_cat_/*I*_L_ = (π*k*_cat_*C_o_t*)^1/2^ (5)

where *I*_cat_ and *I*_L_ represent the catalytic and limiting current in the presence and absence of analytes, respectively. Based on the linear equations of *I*_cat_/*I*_L_-*t*^1/2^ plots in the inset of Figure 5C–F, the *k*_cat_ on GCE, Cu-TCPP/GCE, graphene/GCE, and Cu-TCPP/graphene/GCE is calculated to be 288.9, 1.499 × 10^3^, 5.804 × 10^3^, and 1.635 × 10^4^. The results mean that the electro-oxidation of DA on the Cu-TCPP/graphene/GCE surface has a higher electrocatalytic rate. Some possible reasons are provided: (1) the lamellar structure of Cu-TCPP and graphene nanosheets is beneficial to the accumulation and electron transfer of DA; (2) the Cu^2+^ in Cu-TCPP nanosheets has high catalytic activity for the oxidation of DA; (3) the excellent electrical conductivity of graphene can further improve the electron transfer ability, and then accelerates the electrochemical oxidation of DA. 

In general, the electron transfer ability, the accumulation ability, and the electrocatalytic rate of DA are improved on the surface of the Cu-TCPP/graphene composite. Therefore, the Cu-TCPP/graphene/GCEs show significant signal amplification effects toward DA and have good application in the highly sensitive determination of DA.

### 2.4. Electrochemical Reaction Process of DA on Cu-TCPP/Graphene/GCE

**Figure 7:**
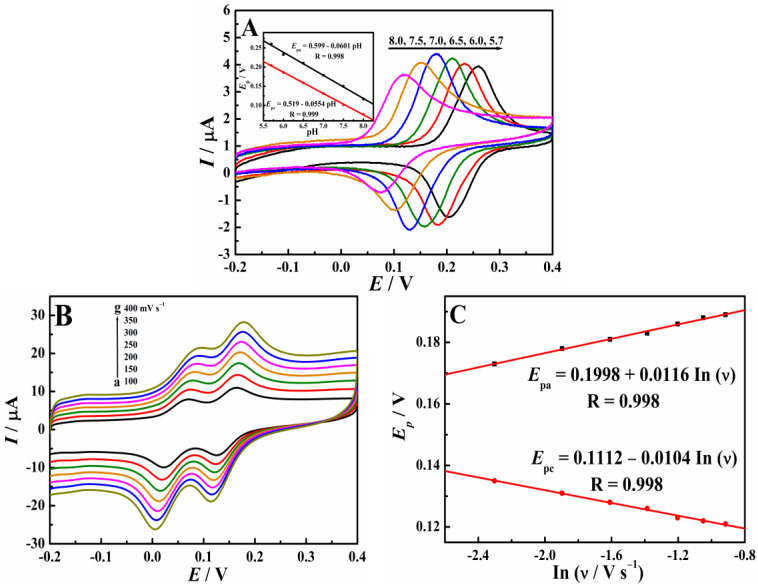
(**A**) CV behaviors of 100 μM DA on Cu-TCPP/graphene/GCE in PBS with different pH values, inset: *E*_pa_-pH plot; (**B**) CV behaviors of 100 μM DA on Cu-TCPP/graphene/GCE in pH 7.0 PBS with different scan rates; (**C**) plots of *E*_p_-ln (*ν*).

Before the voltammetric determination of DA, the influence of pH on the oxidation peak currents and potentials of DA on the Cu-TCPP/graphene/GCE was investigated using CV. As shown in Figure 7A, the oxidation and reduction peak currents (*I*_pa_ and *I*_pc_) are gradually increased with an enlargement of the pH values from 5.7 to 7.0. Then, the signals are decreased as the pH values continue to increase. Therefore, pH 7.0 PBS was selected for the following studies. In addition, the oxidation peak potentials (*E*_pa_) of DA shift negatively, the reduction peak potentials (*E*_pc_) shift positively, and both of them shift linearly with the pH value varying from 5.7 to 8.0, manifesting the protons’ participation in the oxidation process. As shown in the inset of Figure 7A, the linear relationships between *E*_p_ of DA and the pH values are provided. The slopes of the equation are similar to the theoretical value of 0.059 V pH^−1^, suggesting the same amount of transferred electrons and protons participate in the electrochemical reaction of DA on the Cu-TCPP/graphene/GCE.

**Scheme 2:**
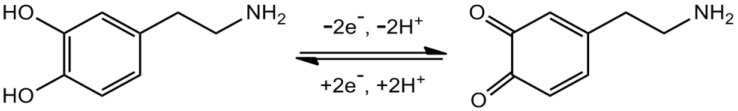
The electrochemical reaction process of DA on Cu-TCPP/graphene/GCE.

In order to further investigate the reaction process of DA, its electrochemical behaviors on the Cu-TCPP/graphene/GCE with different scan rates were studied using CV (Figure 7B). A couple of redox peaks are noticed. The peak currents and the square root of the scan rates show a good linear relationship, indicating DA oxidation is a diffusion-controlled electrode process on the surface of Cu-TCPP/graphene/GCE. Moreover, the redox peak potential (*E*_pa_ and *E*_pc_) moved positively and negatively with the scan rate’s increase. The plot of *E*_p_-ln(ν) is provided in Figure 7C, and a linear relationship is also observed. Based on the Laviron theory [29], the αn value is calculated to be 1.09 and 1.22 for DA according to the slope of the linear equation in the inset of Figure 7C. Here, α is considered as 0.5, so the value of n is 2. In conclusion, the redox of DA on the Cu-TCPP/Ggaphene/GCE is a quasi-reversible process, and two electrons and two protons are involved, which is in keeping with the reported results [34,35]. The proposed redox mechanism is shown in Scheme 2.

### 2.5. Highly Sensitive Determination of DA

**Figure 8:**
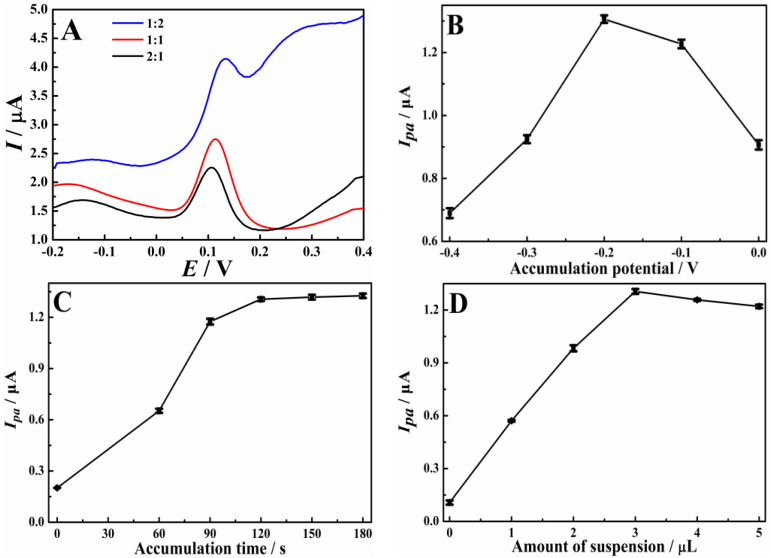

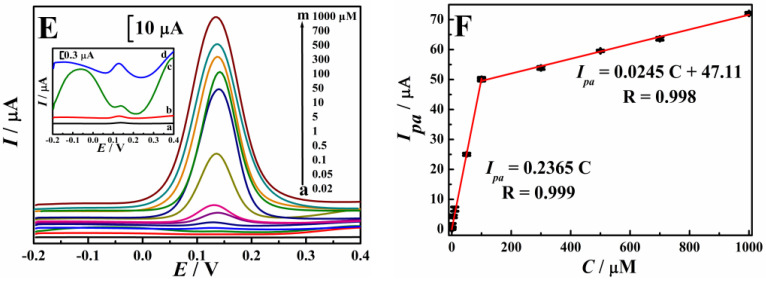
Effects of the volume ratio of Cu-TCPP to graphene (**A**), accumulation potential (**B**), accumulation time (**C**), and the amount of Cu-TCPP/graphene suspension (**D**) on the oxidation response of 1 μM DA, (**E**) DPV curves of DA on the Cu-TCPP/graphene/GCE with varying concentrations, and (**F**) calibration plots. Error bar represents the standard deviation of triple measurements.

Before testing, the experimental conditions were firstly optimized, including the volume ratio of Cu-TCPP to graphene, the accumulation potential, the accumulation time, and the amount of Cu-TCPP/graphene suspension. As shown in Figure 8A, when the volume ratio of Cu-TCPP and graphene is 1:1, the oxidation peak current is the largest. Therefore, equal volumes of Cu-TCPP suspension and graphene suspension were mixed to prepare the Cu-TCPP/graphene composite. Then, the effect of accumulation potential on the oxidation current of DA was discussed (Figure 8B). The increased signals are detected when increasing the potential from −0.4 to −0.2 V. However, when the accumulation potential is further increased to 0 V, the signals start to decrease. This may be a result from the oxidation of DA during the accumulation process at the higher potential. Figure 8C describes the influence of accumulation time on the determination of DA. When the accumulation times are lengthened from 0 to 2 min, the oxidation signals of DA are increased obviously. However, further extending the time to 3 min, slight enhancement is found, indicating the surface reaches a saturation status. In the end, the optimal modification amount of Cu-TCPP/graphene suspension was studied (Figure 8D). A remarkable increase in the oxidation signal of DA is noticed with the volume of modification from 0 to 3 μL, and then it decreased slightly. This may be because of the enhanced accumulation ability resulting from the modification of the Cu-TCPP/graphene composite. To sum up, the best test conditions are as follows: (a) pH value of buffer solution: 7.0, (b) materials mixing volume ratio: 1:1, (c) accumulation potential: −0.20 V, (d) accumulation time: 2 min, and (e) amount of modification: 3 μL.

Under the optimal conditions, the DPV behaviors of different concentrations of DA on the surface of Cu-TCPP/graphene/GCE were recorded, as shown in Figure 8E. The signal increases linearly in the concentration ranges of 0.02–100 μM and 100–1000 μM (Figure 8F). The linear regression equations are provided in the inset of Figure 8F, and the detection limit is calculated to be 3.6 nM for the first linear range based on the triple signal-to-noise ratio. The linear range and LOD of some reported electrochemical sensors were compared with the results obtained on the Cu-TCPP/graphene/GCE (Table 2). Obviously, the Cu-TCPP/graphene/GCE has superior electrochemical sensing properties for the determination of DA.

Then, the repeatability, reproducibility, stability, and selectivity of the Cu-TCPP/graphene/GCE for the voltametric determination of DA were also explored. The repeatability was evaluated by using the same Cu-TCPP/graphene/GCE to perform ten successive DPV measurements of 1 µM DA in the optimized conditions. The relative standard deviation (RSD) was calculated to be 3.7%. The reproducibility was examined by fabricating ten Cu-TCPP/graphene/GCEs independently at the same conditions, and their oxidation signals were recorded. The corresponding RSD value was calculated to be 3.2%. In order to investigate the storage stability of a Cu-TCPP/graphene/GCE, it was stored at 4 °C for seven days. After that, the peak currents were examined, and the values retained 92.6% of their initial values. Therefore, the Cu-TCPP/graphene/GCE is confirmed to own good repeatability, high reproducibility, and strong storage stability for DA sensing applications.

The influences of various potential interferences on the determination of DA were studied to test the selectivity of the Cu-TCPP/graphene/GCE. The electrochemical signals of 1 μM DA on the Cu-TCPP/graphene/GCE were compared with the results in the presence of various interferences. No influence was observed for the determination of 1 μM DA after the addition of 1000 μM ascorbic acid, 100 μM uric acid, or the addition of Na^+^, K^+^, Cu^2 +^, Ca^2+^, Zn^2+^, Mg^2+^, and Fe^2+^ at a concentration level of 2 mM. 

Here, serum samples were selected to evaluate the analytical feasibility and accuracy of the newly developed DA electrochemical sensor based on the Cu-TCPP/graphene/GCE. The samples were taken from the hospital in Xianning City. The recovery test was performed by using the standard addition method. The analysis results are presented in Table 3. The recoveries are in the range of 95.4–105.0, while the relative standard deviations (RSD, *n* = 3) are 1.4–3.4%. The results prove that the Cu-TCPP/graphene/GCE is a reliable electrochemical sensing platform that can be applied for the highly accurate determination of DA in actual samples.

## 3. Materials and Methods

### 3.1. Reagents and Apparatus

Copper(II) nitrate trihydrate (Cu(NO_3_)_2_·3H_2_O), graphite powder (SP), *N*,*N*-dimethylformamide (DMF), *N*-methyl-2-pyrrolidone (NMP), and ethanol were bought from Sinopharm Chemical Reagent Co., Ltd. (Shanghai, China). Tetrakis(4-carboxyphenyl)porphyrin (H_2_TCPP) and dopamine hydrochloride were purchased from Aladdin Chemistry Co., Ltd. (Shanghai, China). High-purity water was used for all the experiments.

The structural properties of the prepared materials were characterized using X-ray diffraction pattern (XRD, X’Pert PRO diffractometer, Cu kα1 radiation, Panalytical Company, Almelo, The Netherlands) and Fourier transform infrared spectroscopy (FT-IR, Equinox-55 Fourier transform infrared spectrometer, Bruker, Karlsruhe, Germany). The morphological properties were studied using scanning electron microscopy (Nova NanoSEM 450 microscope, FEI Company, Eindhoven, The Netherlands) and transmission electron microscopy (Tecnai G2 F30 microscope, FEI Company, Eindhoven, The Netherlands). 

Electrochemical tests were performed on a CHI 660E electrochemical workstation (Shanghai Chenhua Instrument Co., Ltd., Shanghai, China) using a conventional three-electrode system. A Cu-TCPP/graphene/GCE (diameter: 3 mm) as the working electrode, a saturated calomel electrode (SCE) as the reference electrode, and a platinum wire as the counter electrode were used. 

### 3.2. Preparation of Cu-TCPP/Graphene Composite

**Scheme 3:**
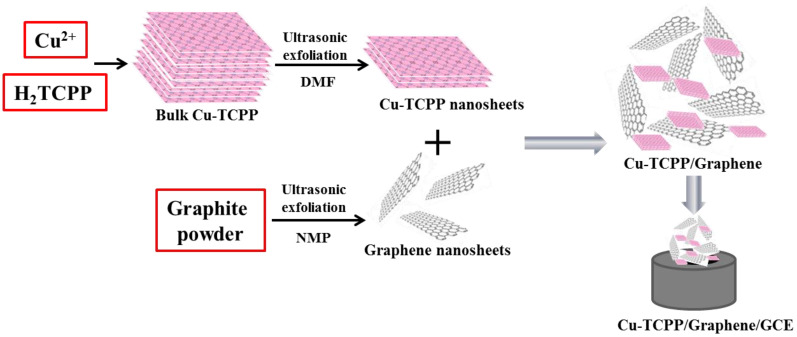
Schematic plot of the construction of Cu-TCPP/graphene/GCE.

As shown in Scheme 3, Cu-TCPP nanosheets and graphene nanosheets were both obtained by ultrasonic exfoliation. Firstly, bulk Cu-TCPP MOFs were prepared based on the reported method [38]. A certain amount of Cu(NO_3_)_2_·3H_2_O (0.2174 g) and H_2_TCPP (0.2372 g) were dissolved in 60 mL of a mixed solvent (V_DMF_:V_ethanol_ = 3:1). A small, capped vial was used for the above mixture, and then placed in a 80 °C oven for 24 h. After that, the mixture was centrifuged, the precipitate was washed using ethanol, and then it was dried in a vacuum oven (60 °C). For the preparation of Cu-TCPP nanosheets, a certain amount of the above obtained Cu-TCPP samples were added into DMF (1 mg/mL). Then, the mixture was placed in an ultrasonic bath (110 W, 40 kHz) for 2 h. Subsequently, the centrifugation technology was used to collect the upper suspension (2000 rpm, 20 min), and the samples were defined as Cu-TCPP nanosheets.

Graphene nanosheets were synthesized using the same ultrasonic exfoliation [39]. Specifically, 30 mL of NMP was used to disperse the graphite powder (600 mg) and sodium citrate (600 mg). After 2 h of ultrasound treatment, the supernatant was obtained as a graphene suspension by centrifugation (2000 rpm, 20 min).

Cu-TCPP/Graphene composite suspension was prepared by mixing the two above-mentioned suspensions in equal volume.

### 3.3. Preparation of Cu-TCPP/Graphene-Composite-Modified GCEs

Before modification, the surface of GCE (diameter: 3.0 mm) was polished using 0.05 μm alumina slurry, and then washed with ethanol and ultrapure water in an ultrasonic bath. Afterwards, 4.0 µL of the Cu-TCPP/graphene suspension was dropped onto the GCE surface and dried with an infrared lamp. The prepared GCE was named Cu-TCPP/graphene/GCE, and then used to construct the DA electrochemical sensing platform (Scheme 3). For comparison, the same programs were carried out to prepare Cu-TCPP/GCE and graphene/GCE using Cu-TCPP and graphene suspensions.

## 4. Conclusions

A simple electrochemical sensor for ultrasensitive determination of DA was developed using the Cu-TCPP/graphene/GCE. The Cu-TCPP/graphene composites feature a remarkable signal enhancement effect toward the oxidation of DA, resulting from its sizeable electroactive surface area, fast electron transfer ability, strong accumulation ability, and high electrocatalytic rate. Throughout the sensing performance of the Cu-TCPP/graphene/GCE, it shows wide linear range, low detection limit, vital reproducibility, good stability, and selectivity for the quantitation of DA. Moreover, the successful applications of this sensor in serum samples indicate promising sensing application potentials.

## Figures and Tables

**Table 1:** The linear regression equations of *Q*_f_-*t*^1/2^ and *Q*_r_-*f*(*t*) on different GCEs.

Electrode	Forward Step	Reverse Step
GCE	*Q*_f_ = 0.7796 + 2.444 *t*^1/2^	|*Q*_r_| = 0.7550 + 2.397 *f*(*t*)
Cu-TCPP/GCE	*Q*_f_ = 1.228 + 4.351 *t*^1/2^	|*Q*_r_| = 1.122 + 4.665 *f*(*t*)
Graphene/GCE	*Q*_f_ = 1.448 + 4.398 *t*^1/2^	|*Q*_r_| = 1.333 + 4.611 *f*(*t*)
Cu-TCPP/Graphene/GCE	*Q*_f_ = 2.174 + 6.697 *t*^1/2^	|*Q*_r_| = 1.766 + 8.402 *f*(*t*)

**Table 2:** Comparison of different modified electrodes for the determination of DA.

Modified Electrode	Linear Range (µM)	LOD (nM)	Ref.
g-C_3_N_4_/MWNTs/GO/GCE	2–100	220	[10]
Cu_x_O-ZnO/PPy/RGO/GCE	0.05–430	10	[11]
Fe_2_O_3_-NiO@GO/GCE	10–1500	5	[12]
f-MWCNTs/GO/AuNPs/AuE	1–400	500	[13]
SnO_2_/α-Fe_2_O_3_ hierarchical nanorods/CPE	0.1–70	40	[36]
Pd@NCF/GCE	0.5–230	107	[37]
Cu-TCPP/Graphene/GCE	0.02–100, 100–1000	3.6	This work

PPy: polypyrrole; AuE: gold electrode; CPE: carbon paste electrode; NCF: nitrogen-enriched carbon frameworks.

**Table 3:** Determination of DA in different serum samples.

Samples	Added (μM)	Found (μM)	Recovery (%)	RSD (%)
1	0.050	0.048	96.0	2.7
	0.20	0.21	105.0	3.1
2	5.00	5.21	104.2	3.2
	100.00	103.40	103.4	2.8
3	50.00	48.90	97.8	2.5
	300.00	286.34	95.4	3.4
4	500.00	487.52	97.5	1.4
	800.00	775.33	96.9	2.3

## Data Availability

Data will be made available upon reasonable request.

## Sample Availability

Samples of the compounds are available from the authors upon reasonable request.
